# Silibinin attenuates radiation-induced intestinal fibrosis and reverses epithelial-to-mesenchymal transition

**DOI:** 10.18632/oncotarget.20624

**Published:** 2017-09-02

**Authors:** Joong Sun Kim, Na-Kyung Han, Sung-Ho Kim, Hae-June Lee

**Affiliations:** ^1^ Research Center, Dongnam Institute of Radiological & Medical Sciences, Busan, Korea; ^2^ Division of Basic Radiation Bioscience, Korea Institute of Radiological and Medical Sciences, Seoul, Korea; ^3^ College of Veterinary Medicine, Chonnam National University, Gwangju, Korea

**Keywords:** silibinin, fibrosis, EMT, TGF-β1, radiation, Pathology Section

## Abstract

Radiotherapy is a common treatment for cancer patients, but its use is often restricted by the tolerance of normal tissue. As cancer patients live longer, delayed radiation effects on normal tissue have become a concern. Radiation-induced enteropathy, including inflammatory bowel disease and fibrosis, are major issues for long-term cancer survivors. To investigate whether silibinin attenuates delayed radiation-induced intestinal injury in mice, we focused on intestinal fibrotic changes. Silibinin improved delayed radiation injuries in mice in association with decreased collagen deposition within the intestines and deceased transforming growth factor (TGF)-β1 levels in the intestine and plasma. Treating mice bearing CT26 mouse colon cancer tumors with both silibinin and radiation stimulated tumor regression more than radiation alone. We also investigated the effect of silibinin on the radiation-induced epithelial-to-mesenchymal transition (EMT), the primary mechanism of fibrosis. We assessed changes in E-cadherin, N-cadherin, and α-smooth muscle actin expression, and demonstrated that silibinin attenuates radiation-induced EMT. Irradiating intestinal epithelial cells increased TGF-β1 levels, but silibinin suppressed TGF-β1 expression by inhibiting Smad2/3 phosphorylation. These results suggest silibinin has the potential to serve as a useful therapeutic agent in patients with radiation-induced intestinal fibrosis.

## INTRODUCTION

While radiation therapy is the most widely used cancer treatment, over 60% of patients who receive pelvic or abdominal therapy exhibit acute bowel toxicity. Acute intestinal complications still occur but are generally transient, whereas chronic gastrointestinal side effects can affect the quality of life [1]. Because the clinical evolution of delayed intestinal toxicity is progressive and inevitable, these complications are of concern in clinical practice [2]. Radiation injury is often mild, but occasionally it may be severe and potentially as malignant as the primary disease. Extensive intestinal resection can manage small bowel injury because limited surgical treatment of chronic radiation enteritis is associated with high late mortalities and high complication rates [3]. Late intestinal toxicity always evolves into a chronic wound-healing process typically associated with mesenchymal cell hyperplasia and activation, tissue disorganization, and fibrillar collagen deposition [4]. Several fibrogenic and inflammatory cytokines, including TGF-β1, promote late radiation injury [5-8]. To date, preventative strategies and novel therapies for radiation enteritis have not reduced the need for surgical intervention [9, 10].

Silibinin is a natural polyphenolic flavonoid extracted from the fruits and seeds of milk thistle (*Silybum marianum*). Silibinin has been used clinically and consumed as a dietary supplement for its anti-hepatotoxic activity in liver disease treatment [11]. Silibinin is well tolerated and largely free of adverse effects. This compound has been shown to be nontoxic in acute and chronic tests, even at large doses, and no LD_50_ for silibinin has been found in animal testing [11, 12]. Silibinin has shown promising and potential anti-tumor efficacy in models of several cancer types, including colon cancer [12-14]. Regarding colon cancer, a study using a rodent model demonstrated that silibinin exerted a preventive effect against azoxymethane-induced colon carcinogenesis [15].

Silibinin inhibits colorectal cancer growth by inhibiting tumor cell proliferation and angiogenic activities. The inhibition of mitogen-activated protein kinase (MAPK) signaling may account for the anti-proliferative and pro-apoptotic effects [16]. The downregulated expression of nitric oxide synthase, cyclooxygenase, hypoxia-inducible factor, and vascular endothelial growth factor might lead to the anti-angiogenic effect of silibinin in colorectal carcinoma [16]. Overall, silibinin has potential applications in treating human colorectal cancer [14]. We examined the potential radioprotective effect of silibinin using radiation-induced intestinal fibrosis mouse models and studied its mechanism *in vivo* and *in vitro*.

## RESULTS

### Silibinin attenuates radiation-induced intestinal fibrosis

**Figure 1:**
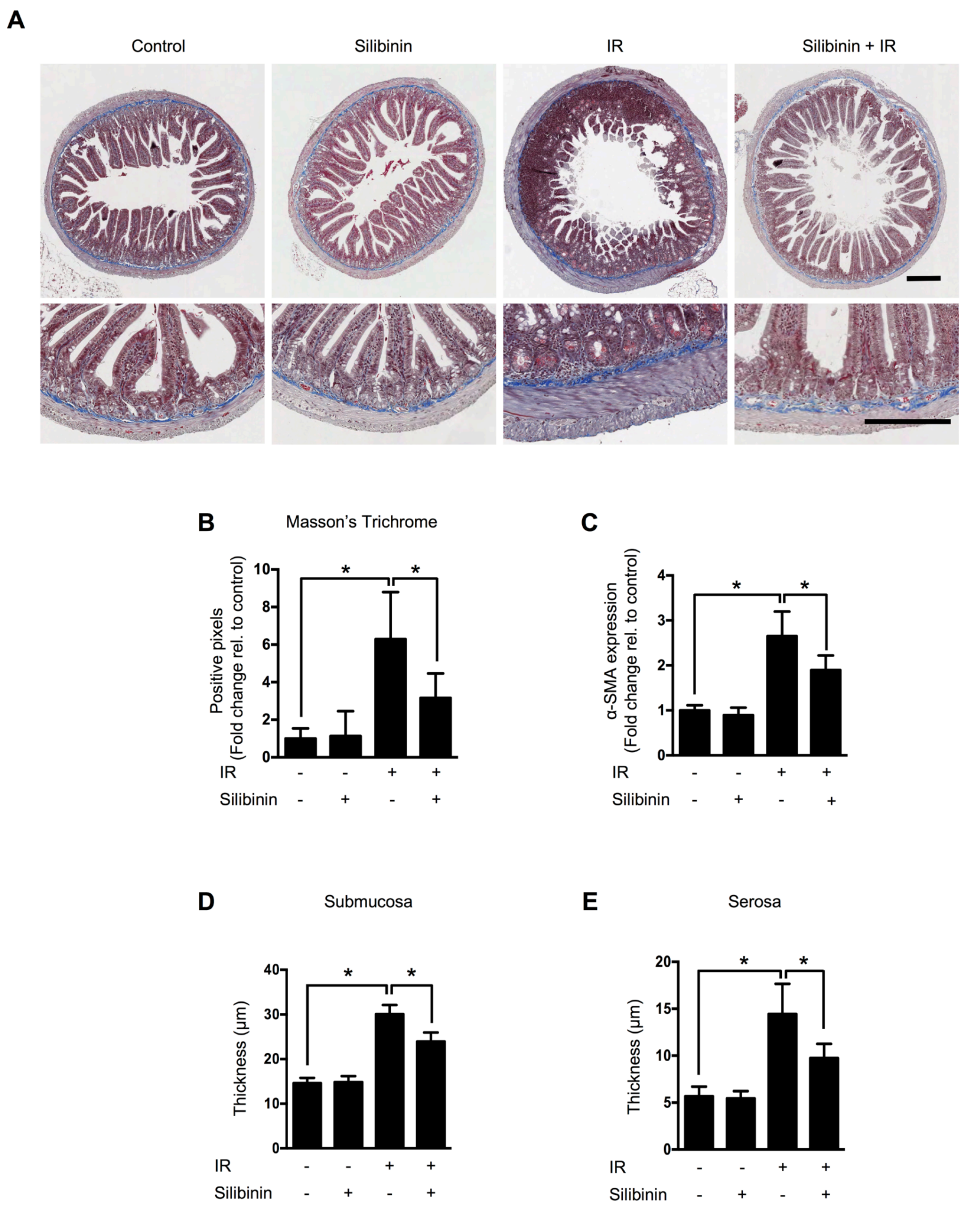
The effect of silibinin on radiation-induced intestinal fibrosis **A.** Representative images of Masson’s trichrome staining, which was performed to detect collagen deposition in jejunum harvested from mice at 200 days after exposure to 13 Gy of partial-body IR. Scale bar = 200 μm. **B.** Quantification of Masson’s trichrome staining. **C.** Real-time qRT-PCR for α-SMA in the intestine. **D.** Measurements of submucosal and **E.** serosal thickness. The data are presented as the mean±SD; *n* = 10, *p < 0.05.

To investigate silibinin’s intestinal effects, we exposed the abdomens of mice to 13 Gy of ionizing radiation (IR) under anesthesia using an X-RAD 320 X-ray system at a dose rate of 2 Gy/min. This was to reduce radiation-induced bone marrow injury using localized abdominal IR [17]. At 200 days after radiation, we sacrificed animals and analyzed intestinal fibrosis. No mortality was observed in any group over the course of 200 days. Intestinal fibrosis was detected by Masson’s trichrome (MT) staining (Figure 1A). Fibrotic areas were characterized by a complete loss of intestinal structure, which was replaced by dense extracellular matrix deposition and intense luminal inflammatory cell infiltration. A reduction of 50% (*p* < 0.05) in the deposition of collagen (blue) in the submucosa and muscle surrounding the intestine was observed in irradiated mice treated with silibinin compared with vehicle-treated mice (Figure 1B).

Confirming the correlation with the MT staining results, upregulated mRNA levels of α-smooth muscle actin (α-SMA), a fibrosis marker, were observed using real-time quantitative reverse transcription PCR (qRT-PCR). Silibinin treatment inhibited α-SMA upregulation by 28% (*p* < 0.05) compared with exposure to radiation alone (Figure 1C). In the intestine sections from irradiated mice treated with vehicle, the submucosal and serosal (muscle) layer thicknesses were increased from 14.6±1.2 to 30.1±2.1 μm and from 5.7±1.0 to 14.4±3.2 μm, respectively, compared with the control mice and the irradiated mice (*p* < 0.05). Silibinin treatment reduced these histological changes, by 22% (*p* < 0.01) and 32% (*p* < 0.01), respectively, compared with the mice exposed to radiation alone (Figure 1D, 1E).

### Silibinin downregulates TGF-β1 levels in tissue and blood

**Figure 2:**
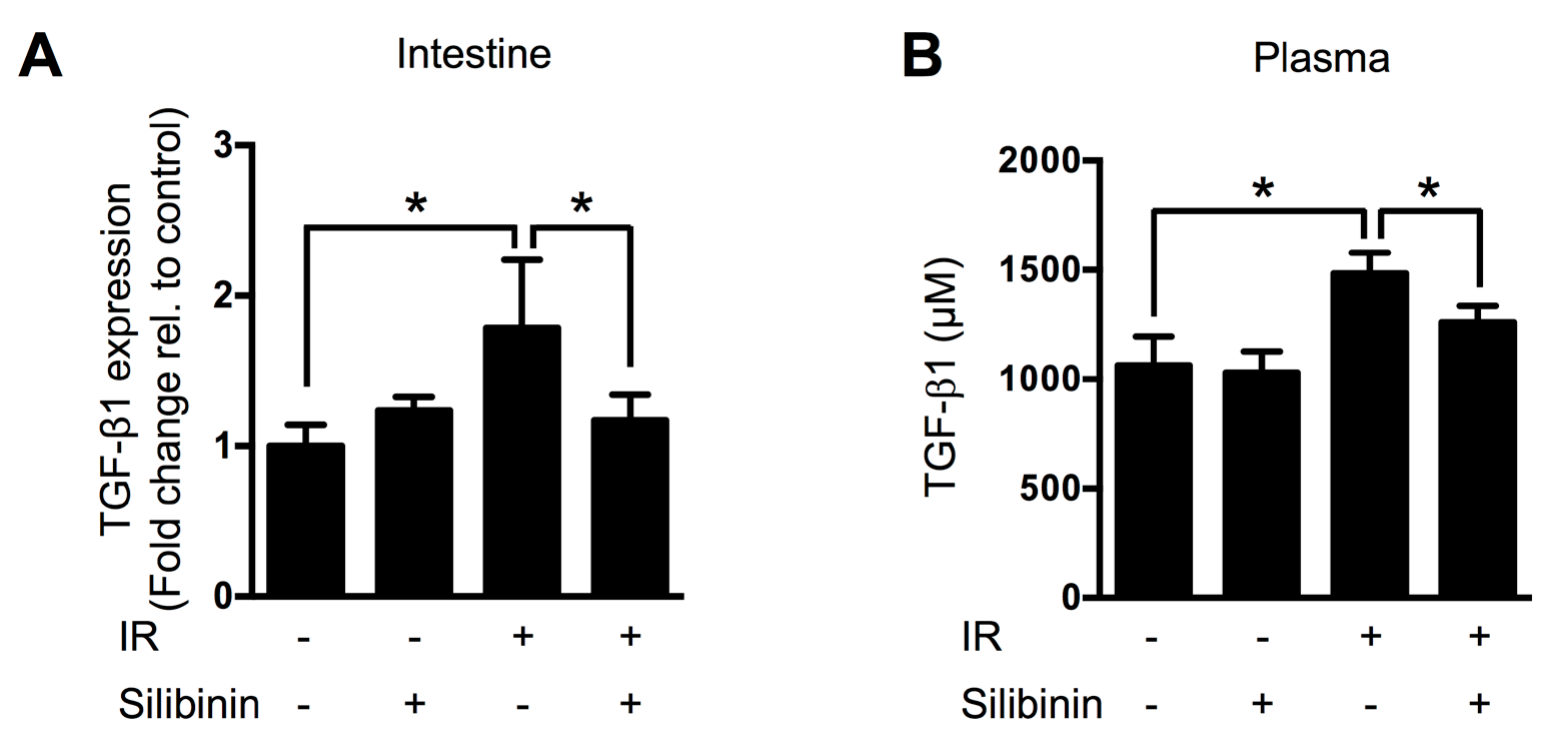
TGF-β1 levels in the intestine and plasma of animals at 200 days after IR **A.** Real-time qRT-PCR results showing TGF-β1 mRNA levels and **B.** ELISA results showing soluble TGF-β1 protein levels. The data are presented as the mean±SD; *n* = 10, *p < 0.05.

TGF-β1 is known as a key cytokine that stimulates radiation-induced fibrosis. We investigated whether TGF-β1 participates in delayed radiation enteropathy by performing real-time qRT-PCR for intestinal TGF-β1 in each group (*n* = 6) at 200 days after IR. Compared with control, exposure to 13 Gy of IR upregulated TGF-β1 expression in the jejunum. Silibinin downregulated TGF-β1 expression in the jejunum by 34% (*p* < 0.05) compared with the irradiated control (Figure 2A). We also measured plasma TGF-β1 levels by ELISA, and found that silibinin inhibited TGF-β1 release from the fibrotic region into the blood by 15% (*p* < 0.05) compared with the irradiated control (Figure 2B).

### Silibinin ameliorates radiation-induced EMT

**Figure 3:**
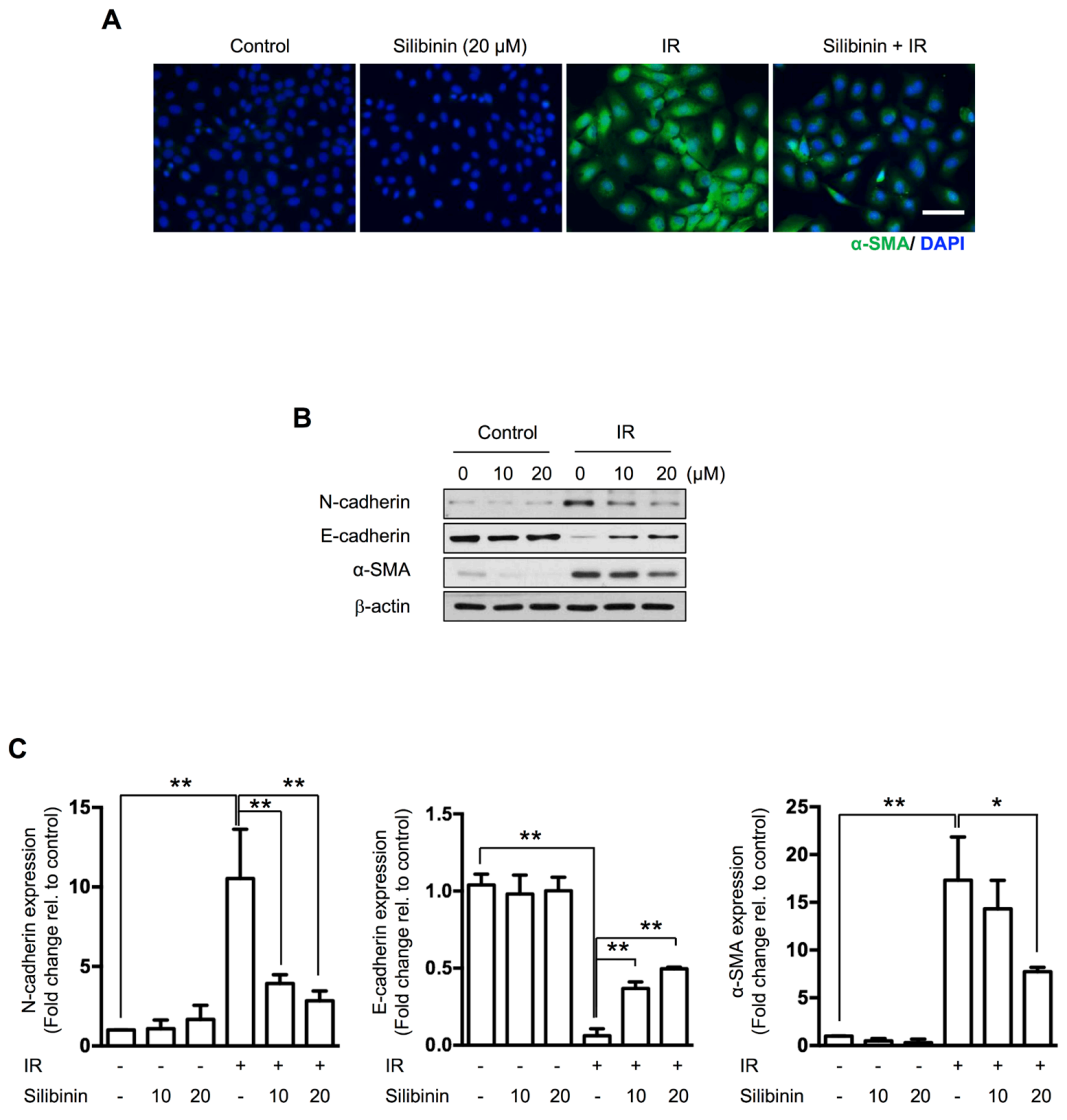
The effect of silibinin on radiation-induced EMT in IEC6 cells **A.** Immunofluorescence of α-SMA and counterstaining with DAPI. IEC6 cells were treated with 20 μm silibinin 3 h prior to 10 Gy of IR and incubated for 72 h. Scale bar = 50 μm. **B.** Western blotting for N-cadherin, E-cadherin and α-SMA in irradiated IEC6 cells and **C.** quantification. The data are shown as the mean±SD; *n* = 3, *p < 0.05 and **p < 0.01.

To investigate the effect on silibinin in EMT of the intestine, we performed immunofluorescence for α-SMA in IEC6 rat intestinal epithelial cells. Radiation increased cytoplasmic α-SMA expression, and this was decreased by silibinin (Figure 3A). Western blotting confirmed that silibinin treatment ameliorated radiation-induced EMT represented by inhibition in phenotype changes, loss of the epithelial marker E-cadherin, and gain of the mesenchymal marker N-cadherin (Figure 3B, 3C). We also determined cytotoxicity and the effect on cell proliferation of silibinin in IEC6 cells using LDH and MTT assays. No cytotoxic or anti-proliferation effects were observed in IEC6 cells treated with up to 20 µM silibinin (Supplementary Figure 1).

### Silibinin suppresses radiation-induced TGF-β1 and its signaling pathway

**Figure 4:**
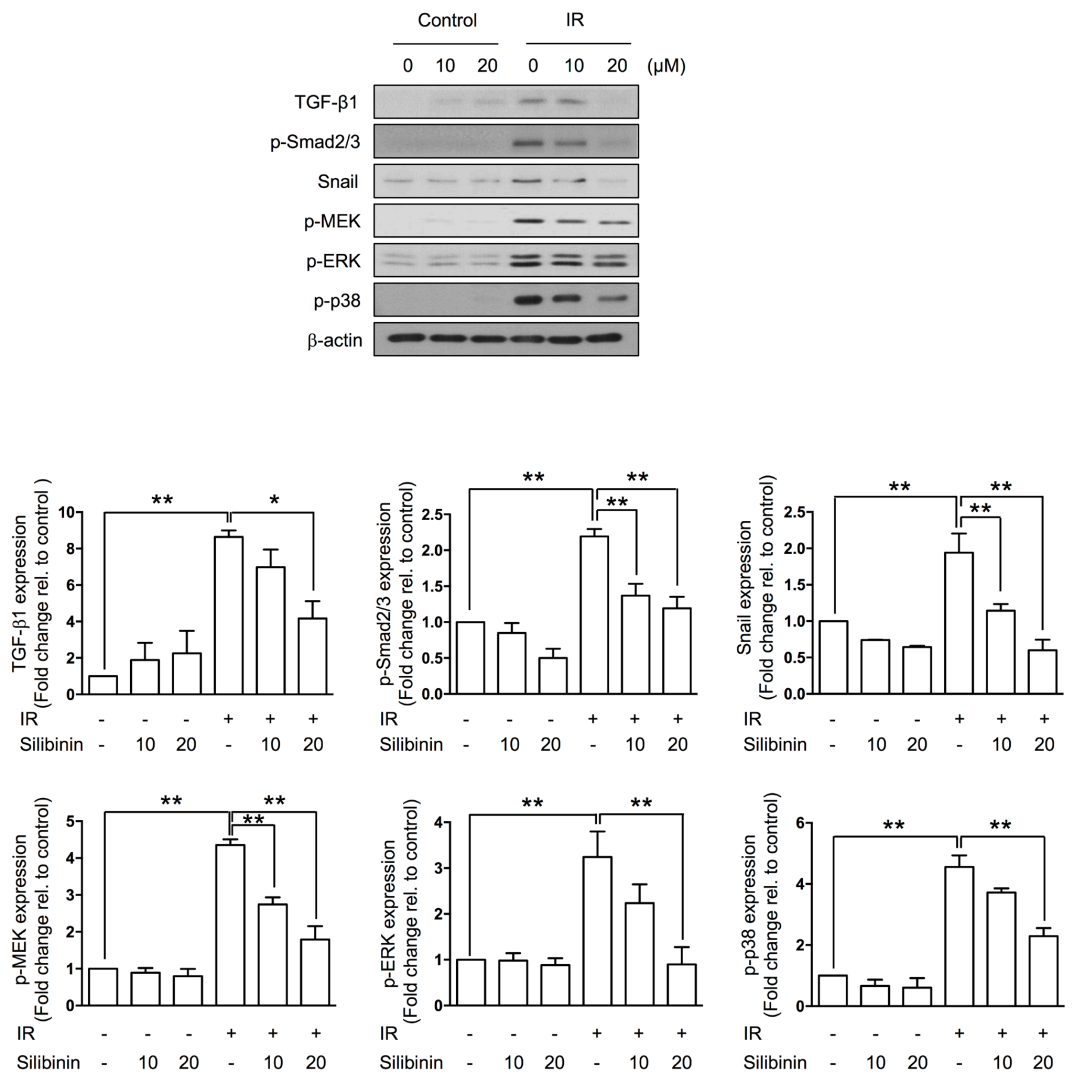
Silibinin suppresses TGF-β1 and the related signal pathway against IR Representative western blots and quantification for TGF-β1, p-Smad2/3, Snail, p-MEK, p-ERK, p-p38, and β-actin. Note that silibinin inhibited radiation-induced EMT signaling in a dose-dependent manner. The data are shown as the mean±SD; *n* = 3, *p < 0.05 and **p < 0.01.

Because radiation is known to evoke multiple pathways involved in the EMT, we investigated TGF-β1 expression and TGF-β1-associated signaling in IEC6 cells. While the radiation upregulated TGF-β1 and Snail expression, it increased phosphorylation Smad2/3. The silibinin treatment inactivated these TGF-β1-associated signals and attenuated extracellular signal-regulated kinase (ERK) and p38 MAPK activation (Figure 4).

### Silibinin directly inhibits TGF-β1-induced EMT via p-Smad2/3 inactivation

**Figure 5:**
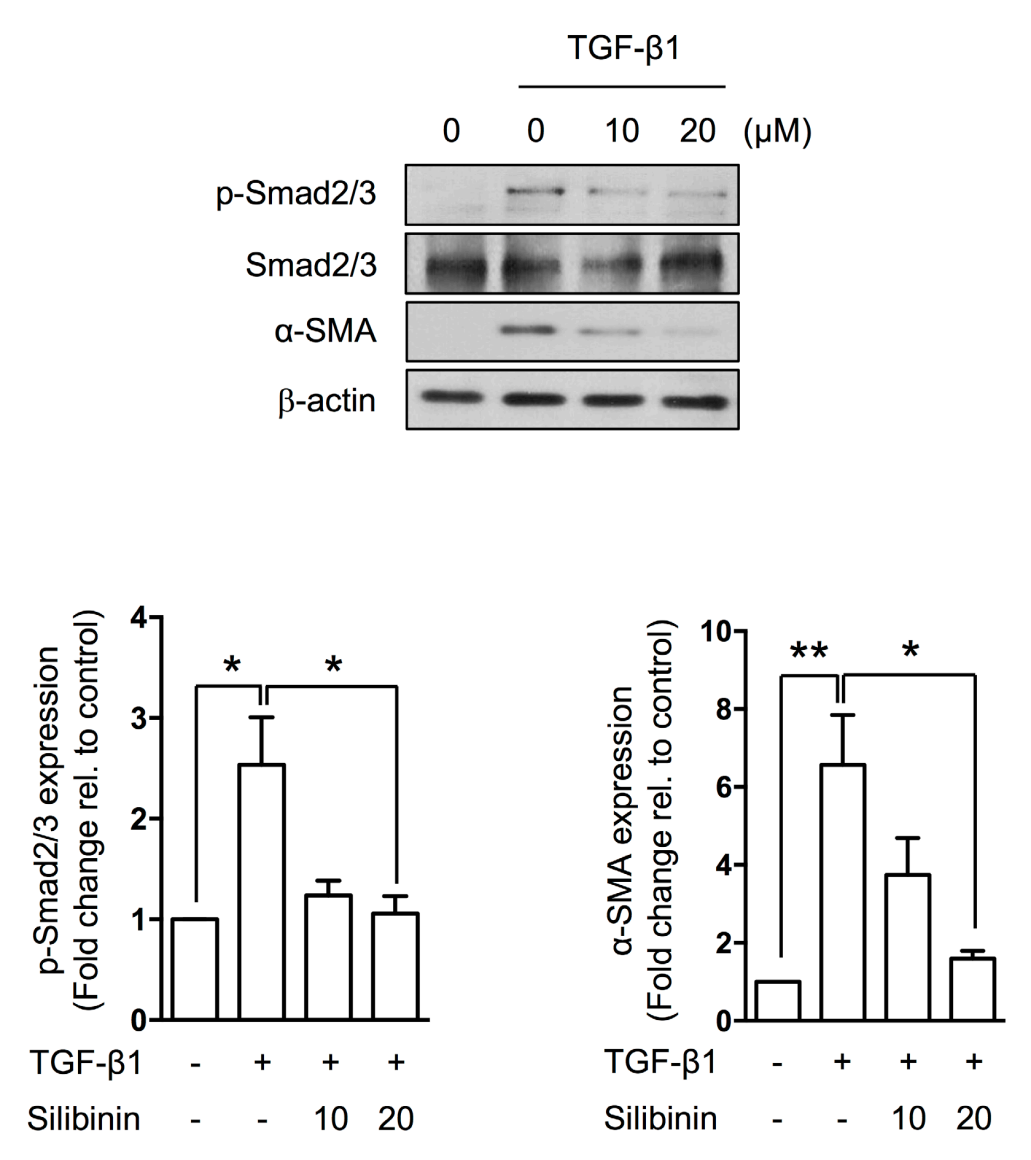
Suppression of TGF-β1-induced EMT by silibinin Representative western blots for p-Smad2/3, Smad2/3, α-SMA, and β-actin. The data are shown as the mean±SD; *n* = 3, *p < 0.05 and **p < 0.01.

To investigate the effect of silibinin on TGF-β1-induced EMT, we treated IEC6 cells with silibinin for 3 h before treatment with 10 ng/ml human TGF-β1 for 48 h. We observed a cellular phenotype transition and upregulated p-Smad2/3 expression. Silibinin prevented the phosphorylation of Smad2/3 by TGF-β1 (Figure 5).

### Early phase silibinin treatment of radiation injury suppresses TGF-β1 and EMT of the intestine

**Figure 6:**
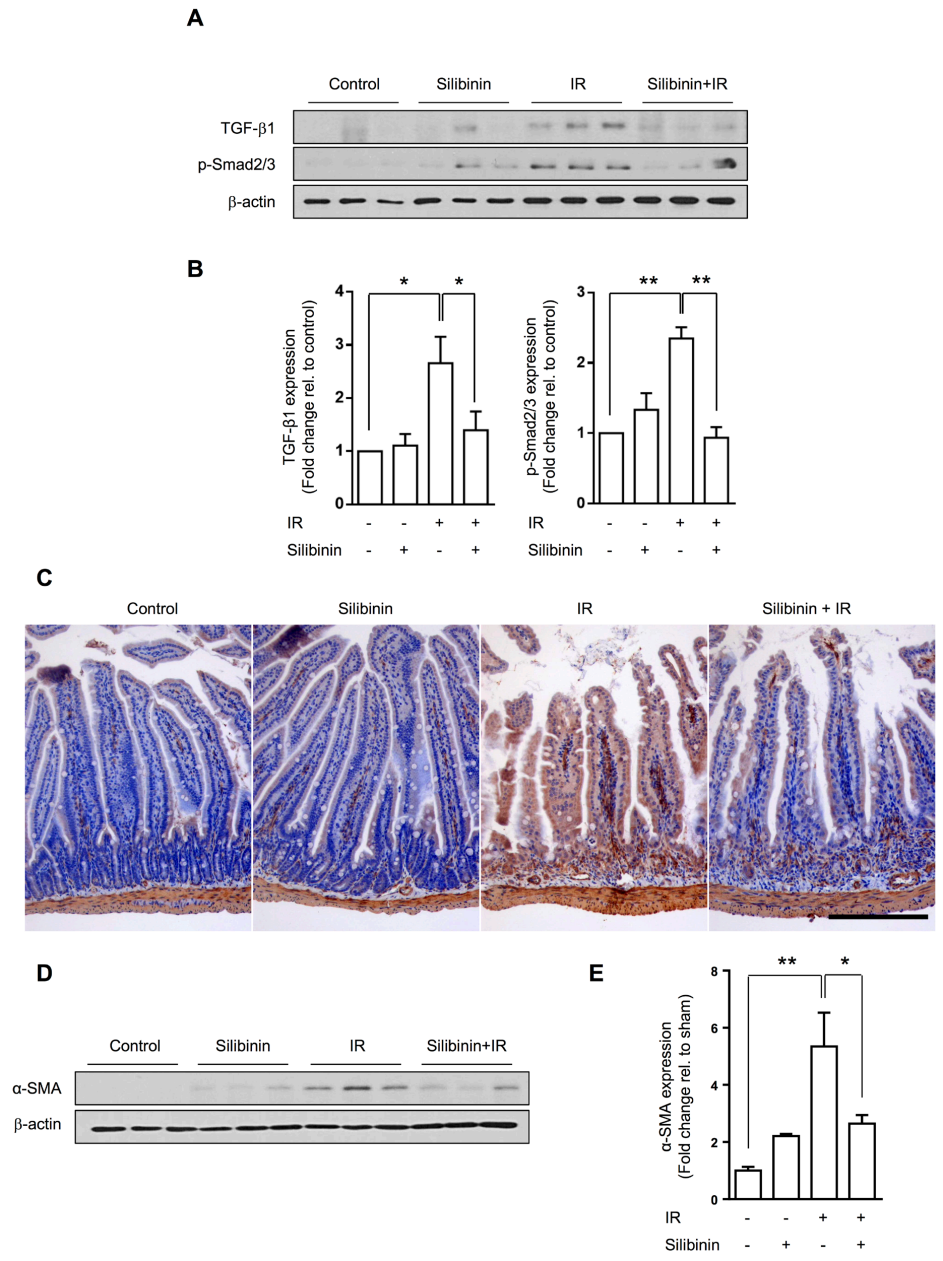
Effect of silibinin in the early phase of radiation-induced intestinal injury **A.** TGF-β1 and p-Smad2/3 expression in the intestine following silibinin and/or radiation in western blots. Intestines were harvested from mice administered 3 doses of oral silibinin (100 mg/kg) and/or 13 Gy of IR at 12 h after IR. **B.** Quantified TGF-β1 and p-Smad2/3 expression. **C.** Representative image of α-SMA in the intestine harvested from mice administered 6 doses of oral silibinin (100 mg/kg) and/or 13 Gy of IR at 3.5 days after IR. Brown color indicates positive staining for α-SMA. Scale bar = 200 μm. **D.** Western blots for α-SMA in the intestine and **E.** quantification. The data are presented as the mean±SD; *n* = 3, *p < 0.05, **p < 0.01.

To determine the effect of early phase silibinin in radiation-induced intestinal injury, we analyzed the intestine at 12 h with 3 doses, and at 3.5 days with 6 doses. Treatment of silibinin and the experimental procedure are described in supplemental information. At 12 h after irradiation, no fibrotic changes were observed in the intestine. Silibinin had no protective effect in radiation-induced apoptotic death as evaluated by TUNEL assays (Supplementary Figure 2A, B). At 3.5 day after irradiation, intestinal cell survival decreased, as shown by immunohistochemistry of Ki67 and numbers of crypts and length of villi in the intestine. Silibinin showed no protective effect on intestinal cell survival (Supplementary Figure 2C-E). Although silibinin did not prevent radiation-induced cell death, silibinin inhibited TGF-β1 and p-Smad2/3 upregulation (*p* < 0.05 and *p* < 0.01, respectively) at 12 h after IR, as well as α-SMA expression (*p* < 0.05) at 3.5 days after IR (Figure 6).

### Silibinin does not interfere with the therapeutic effect of radiation on cancer

**Figure 7:**
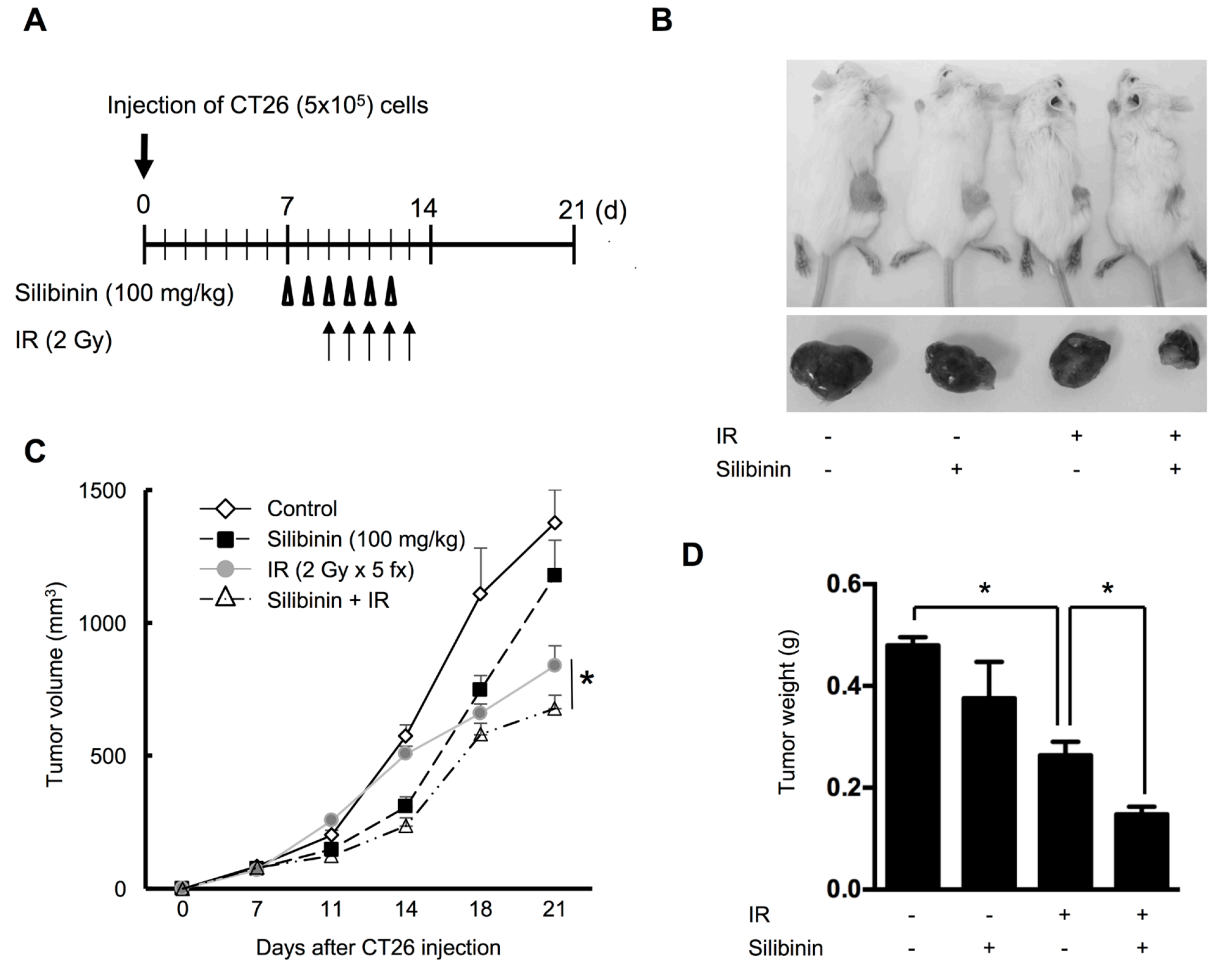
The effect of silibinin on radiotherapy **A.** Protocol diagram for silibinin treatment and fractionated irradiation. The effect of silibinin on tumor growth was measured in CT26 allografts in BALB/c mice. Six silibinin doses (100 mg/kg) were administered orally to tumor-bearing mice before and after IR (2 Gy × 5 fractionations for 5 days). **B.** Growth curve of the CT26 tumor model following silibinin and/or IR treatment. Tumor regression in each group is shown in the representative photo **C.** and graph of tumor weight **D.** The data are presented as the mean±SD; *n* = 8, *p < 0.05.

The clinical implications of silibinin combined with radiation were assessed *in vivo* using a CT26 xenograft model. Tumor-bearing mice were sham-irradiated or exposed to a total dose of 10 Gy, and were treated with vehicle or silibinin at a 100 mg/kg per day (Figure 7A). All treatments were well tolerated; no signs of toxicity were observed, and no gross pathological abnormalities were noted during necropsy. Tumors were smaller after exposure to fractionated radiation than to sham IR (*p* < 0.01 at 21 days after tumor implantation) (Figure 7B). Tumors exposed to IR and treated with silibinin exhibited the best tumor regression (*p* < 0.05) and growth inhibition among the groups, but there were no differences between the vehicle- and silibinin-treated groups (Figure 7C, 7D).

The sensitivity of colon cancer cells to IR was assessed using a clonogenic survival assay *in vitro.* We found that 20 μm of silibinin alone reduced the survival fraction compared with the non-treatment control (*p* < 0.05). The combination of 2 Gy irradiation and 20 μm of silibinin reduced the survival fraction compared to that of each single treatment (*p* < 0.05, Supplementary Figure 3).

## DISCUSSION

Radiation is widely used in cancer therapy to achieve local tumor control [1, 2]. Approximately 50% of people with cancer are treated with radiation therapy. While this treatment exerts anti-proliferative and cytotoxic effects in tumor tissue, cytotoxicity in normal tissue remains the most important obstacle to treating cancer without complications [3-5]. Delayed radiation enteropathy is a common radiation therapy-related adverse effect. The progressive condition not only has few available therapeutic options, but also can lead to substantial long-term morbidity and mortality. Although the application of radioprotectors or mitigators can improve the quality of life, very few normal tissue protectors are in clinical use.

In this study, we demonstrated the protective effect of silibinin in radiation-induced intestinal fibrosis. Silibinin inhibited fibrotic changes in the intestine by downregulating TGF-β1 and inhibiting EMT. Silibinin directly inhibited TGF-β1 upregulation and inactivated p-Smad2/3, a downstream effector of TGF-β1 and an EMT stimulator. These changes contributed to EMT suppression, subsequently improving the therapeutic effect of silibinin on delayed radiation injury. In addition, silibinin also facilitated the IR-induced elimination of cancer cells in a CT26 tumor model.

Our results indicate that silibinin inhibits radiation-induced TGF-β1 upregulation in the intestine and blood. Silibinin also suppressed fibrotic changes, which are indicative of radiation enteropathy [18, 19]. TGF-β1 promotes the pathogenesis of radiation-induced intestinal fibrosis [3, 6, 10, 20]. The TGF-β1 mRNA levels in fibroblasts present in fibrotic regions are consistent with the *in vivo* findings of radiation-induced dermal fibrosis studies [21, 22]. Intense TGF-β1 mRNA expression in areas of histopathological injury is especially pertinent to the development of intestinal fibrosis. Our findings of sustained TGF-β1 mRNA expression in the region of intestinal fibrosis and in the plasma are consistent with the results of *in vivo* studies of radiation-induced fibrosis in the lungs, kidneys, and dermis.

The most important finding of this study is that silibinin not only inhibits TGF-β1 signaling, which is a master molecule in the EMT, but also reduces the EMT in TGF-β1-stimulated IEC6 cells. In this study, silibinin suppressed the activation of a complex network of pathways, including the Smad2/3, Snail, and MAPK signaling pathways. TGF-β1 target genes are controlled via Smad2/3-dependent transcriptional regulation. In particular, p-ERK and p-p38 MAPK have been demonstrated to inhibit Smad-independent TGF-β1 responses in intestine epithelial cells. Silibinin decreased the phosphorylation of ERK, p38, and MEK; therefore, silibinin could synergistically suppress the EMT and chronic fibrosis in the intestine. Moreover, silibinin treatment reversed the EMT in IEC6 cells following exposure to radiation and TGF-β1. These results demonstrate the potential use of silibinin to suppress intestinal fibrosis.

Silibinin has been used as a medicinal herb for treating liver cirrhosis, chronic hepatitis, and gallbladder disorders without toxicity. In the present study, daily oral administration of silibinin (100 mg/kg) for 6 days had no toxicity, consistent with the results of Singh *et al.* (100 mg/kg for 7 weeks) [23], and Agarwal *et al.* [24] (up to 2000 mg/kg for 16 days). The repeated oral administration of silibinin has also been reported to be safe in colorectal carcinoma patients (daily doses up to 1.44 g for a week) [25]. Given that silibinin consumption is safe, silibinin has strong translational preventive and therapeutic implications in treating cancer patients. We have evaluated the radiosensitizing properties of silibinin in an *in vivo* model using colon cancer tumor-bearing mice. Consistent with other research results, silibinin showed a partial cytotoxic effect on these cancer cells and exhibited an additive effect in mouse tumors when combined as a pretreatment with IR.

Silibinin had no beneficial effects *in vivo* during the early phase even if we expected silibinin to exert protective effects, such as anti-apoptosis effects or crypt cell survival. Nambiar *et al.* [26] also reported that silibinin had a radiosensitizing effect in prostate cancer while countering IR-induced toxicity in normal tissue. We speculate that the radiation dose and organ-specific sensitivity may explain these differences from our results; 13 Gy of IR may be too high of a dose to protect normal intestinal stem cells against direct radiation-induced cell death in the acute phase. Nonetheless, silibinin prevented radiation-induced TGF-β1 activation and p-Smad2/3 upregulation in the acute phase. Although radiation fibrosis is a chronic and progressive event, TGF-β1 induces EMT in early phases of the disease [27]. Therefore, silibinin treatment in the early phase of radiation injury could still produce anti-fibrotic effects in the intestine in the chronic phase.

We found that silibinin suppresses intestinal fibrosis without disrupting therapeutic radiation by blocking TGF-β1 signaling. Silibinin could potentially be used for safe clinical applications when administered orally.

## MATERIALS AND METHODS

### Animals

Female 6-week-old C57BL/6 and BALB/c mice were purchased from Central Lab. Animal Inc. (Seoul, Korea), and the experiments were performed after 1 week of quarantine and acclimatization. The animals were maintained in a room at 23±2°C with a relative humidity of 50±5%, artificial lighting from 08:00-20:00, and 13-18 air changes per hour. The mice were fed a standard animal diet. All protocols in this study were approved by the Institutional Animal Care and Use Committee of the Korean Institute of Radiological and Medical Sciences (IACUC permit number: KIRAMS2014-0045).

### Radiation

Each mouse was anesthetized with tiletamine/zolazepam (Zoletil 50^®^, Virak Korea, Seoul, Korea), and exposed to 13 Gy of IR using an X-RAD 320 system (Precision X-ray, Inc., North Branford, CT, USA) at 250 kV, 10 mA with 420 mm aluminum added filtration at a dose rate of 2 Gy/min. The radiation field size was 3 cm x 10 cm, which began from the end of the sternum in each mouse. Sham-irradiated mice were treated in exactly the same manner as the irradiated animals but were not irradiated. Silibinin (Sigma-Aldrich, St. Louis, MO, USA) was administered at an optimal dose (100 mg/kg) for radioprotection [15, 16]. Silibinin was dissolved in 0.9% saline for oral administration.

### Measurement of fibrotic intestinal changes

The 40 female C57BL/6 mice were divided into four groups (*n* = 10 per group) as follows: a control group, a radiation group, and two groups that were administered silibinin. Silibinin (100 mg/kg) was administered to mice orally once per day for six days before and after IR. Animals were sacrificed at 200 days after IR, and intestinal tissues were longitudinally fixed in 4% formalin for 24 h and embedded in paraffin wax. Fibrosis was evaluated by examining the area of collagen deposition in intestine sections (n≥10) stained with Masson’s trichrome (MT). Collagen deposition (blue staining) was quantified using Image-Pro Plus image analysis software (Media Cybernetics, Bethesda, MD, USA).

The mRNA levels of α-SMA and TGF-β1 were measured using real-time qRT-PCR. Total RNA was extracted from small intestine tissue using an ISOGEN kit (Nippon Gene, Tokyo, Japan). The α-SMA and TGF-β1 mRNA levels were normalized to GAPDH mRNA and reported as a ratio relative to the mean value obtained for the intestinal tissue of sham-operated mice. PCR primer sequences are in Supplementary Table 1. Plasma was obtained by whole-blood centrifugation at 8900 × g and 4°C for 5 min, then stored at -80°C. The plasma TGF-β1 level (μM) was measured by ELISA (R&D Systems, MN, USA).

### Cell culture and treatment

The IEC6 rat intestinal epithelial and CT26 mouse colon cancer cell lines were obtained from the Korean Cell Line Bank (Seoul, Korea). IEC6 cells were cultured in Dulbecco’s modified Eagle’s medium (WELGENE, Daegu, Korea), and CT26 cells were cultured in RPMI 1640 medium supplemented with 10% fetal bovine serum and antibiotics. The cells were treated with radiation (10 Gy) and/or different concentrations of silibinin (5-100 μM) dissolved in DMSO. The cells were treated with silibinin 3 h before radiation or TGF-β1 (10 ng/ml) treatment and incubated for 48-72 h, depending upon the experiment.

### Western blot analysis

Cells were lysed with RIPA buffer (25 mM Tris-HCl, pH 7.4, 1 mM EDTA, 137 mM NaCl, 1% Triton X-100, 1% sodium deoxycholate, 0.1% SDS) for 30 min. The lysate was centrifuged and quantitated with Bradford assay reagent (Bio-Rad, Hercules, CA, USA) following the manufacturer’s protocol. Intestine tissues were physically minced in Pro-prep^®^ solution (17081, Intron, Sungnam, Gyeonggi-do, Korea), and then proteins were extracted from the lysates. Western blot analysis was performed using the following antibodies: TGF-β1 (1:1000, #3711, Cell Signaling Technology, Danvers, MA, USA), N-cadherin (1:1000, ab18203, Abcam, Cambridge, UK), α-SMA (1:2000, ab5694, Abcam), E-cadherin (1:1000, BD610181, BD Biosciences, Franklin Lakes, NJ, USA), p-Smad2/3 (1:1000, #8828, Cell Signaling Technology), Smad2/3 (1:1000, #3102, Cell Signaling Technology), Snail (1:1000, #3895, Cell Signaling Technology), p-MEK (1:1000, #9154, Cell Signaling Technology), p-ERK (1:1000, #9101, Cell Signaling Technology), p-p38 (1:1000, #9211, Cell Signaling Technology), and β-actin (1:5000, A5316, Sigma-Aldrich, St. Louis, MO, USA). The blots were incubated with those antibodies overnight at 4°C. Protein bands were visualized by electrochemiluminescence. Protein expression was quantified using a Fluor-S Multimager system (Bio-Rad, USA).

### Immunofluorescence analysis

IEC6 cells were cultured on cover slides and then treated with silibinin for 3 h before 10 Gy irradiation. After 72 h, the cells were fixed with 10% formaldehyde and treated with 0.5% Triton X-100 solution. The cells were blocked with 0.5% BSA and then incubated with α-SMA (1:250, ab5694, Abcam) overnight at 4°C. Next the cells were incubated with FITC-conjugated secondary antibody (1:500, ab150077, Abcam) for 2 h in a 4°C chamber. After DAPI counterstaining, the cells were observed under a fluorescence microscope (Axio Imager M2, Carl Zeiss, Jena, Germany).

### CT26 colon tumor models

BALB/c mouse CT26 colon cancer cells (Korean Cell Line Bank, Seoul, Korea) were cultured in RPMI medium containing 10% FBS and 1% antibiotics (penicillin, gentamicin and streptomycin; Gibco BRL, Life Technologies Pty. Ltd., Victoria, Australia). CT26 cells (5 × 10^5^ cells per animal in 100 μl of PBS) were injected subcutaneously into the right flank of each BALB/c mouse. After acclimatization, the mice were randomly divided into the following four groups (*n* = 8 mice/group): the control group, the irradiated group, the silibinin treatment group, and the group subjected to IR and treatment with silibinin. Silibinin (100 mg/kg) was administered to mice for 6 consecutive days beginning 2 days before IR. Tumor volume was measured at 7, 11, 14, 18, and 21 days after implantation using calipers. When the tumors reached a mean volume of approximately 100 mm^3^ (day 7), the mice received sham or partial-body IR. Anesthetized mice were positioned on a tray with the cancer lesion in the radiation field. Partial-body IR was administered at a daily dose of 2 Gy for 5 days. The sham-irradiated mice underwent similar procedures without radiation.

### Statistical analysis

All statistical analyses were performed with Prism 6 (GraphPad software, San Diego, CA, USA). The data were analyzed using one-way ANOVA followed by Tukey’s post hoc test for multiple comparisons. In all cases, *p* values < 0.05 were considered significant.

## SUPPLEMENTARY MATERIALS FIGURES AND TABLE



## References

[1] Andreyev J (2005). Gastrointestinal complications of pelvic radiotherapy: are they of any importance?. Gut.

[2] Hauer-Jensen M, Wang J, Denham JW (2003). Bowel injury: current and evolving management strategies. Semin Radiat Oncol.

[3] DeCosse JJ, Rhodes RS, Wentz WB, Reagan JW, Dworken HJ, Holden WD (1969). The natural history and management of radiation induced injury of the gastrointestinal tract. Ann Surg.

[4] Vozenin-Brotons MC, Milliat F, Sabourin JC, de Gouville AC, Francois A, Lasser P, Morice P, Haie-Meder C, Lusinchi A, Antoun S, Bourhis J, Mathe D, Girinsky T, Aigueperse J (2003). Fibrogenic signals in patients with radiation enteritis are associated with increased connective tissue growth factor expression. Int J Radiat Oncol Biol Phys.

[5] Hauer-Jensen M, Richter KK, Wang J, Abe E, Sung CC, Hardin JW (1998). Changes in transforming growth factor beta1 gene expression and immunoreactivity levels during development of chronic radiation enteropathy. Radiat Res.

[6] Zheng H, Wang J, Hauer-Jensen M (2000). Role of mast cells in early and delayed radiation injury in rat intestine. Radiat Res.

[7] Wang J, Zheng H, Sung CC, Richter KK, Hauer-Jensen M (1998). Cellular sources of transforming growth factor-beta isoforms in early and chronic radiation enteropathy. Am J Pathol.

[8] Waddell BE, Rodriguez-Bigas MA, Lee RJ, Weber TK, Petrelli NJ (1999). Prevention of chronic radiation enteritis. J Am Coll Surg.

[9] Hauer-Jensen M, Denham JW, Andreyev HJ (2014). Radiation enteropathy—pathogenesis, treatment and prevention. Nat Rev Gastroenterol Hepatol.

[10] Wellington K, Jarvis B (2001). Silymarin: a review of its clinical properties in the management of hepatic disorders. BioDrugs.

[11] Flora K, Hahn M, Rosen H, Benner K (1998). Milk thistle (Silybum marianum) for the therapy of liver disease. Am J Gastroenterol.

[12] Singh RP, Dhanalakshmi S, Agarwal C, Agarwal R (2005). Silibinin strongly inhibits growth and survival of human endothelial cells via cell cycle arrest and downregulation of survivin, Akt and NF-kappaB: implications for angioprevention and antiangiogenic therapy. Oncogene.

[13] Singh RP, Deep G, Chittezhath M, Kaur M, Dwyer-Nield LD, Malkinson AM, Agarwal R (2006). Effect of silibinin on the growth and progression of primary lung tumors in mice. J Natl Cancer Inst.

[14] Ravichandran K, Velmurugan B, Gu M, Singh RP, Agarwal R (2010). Inhibitory effect of silibinin against azoxymethane-induced colon tumorigenesis in A/J mice. Clin Cancer Res.

[15] Singh RP, Gu M, Agarwal R (2008). Silibinin inhibits colorectal cancer growth by inhibiting tumor cell proliferation and angiogenesis. Cancer Res.

[16] Li L, Gao Y, Zhang L, Zeng J, He D, Sun Y (2008). Silibinin inhibits cell growth and induces apoptosis by caspase activation, down-regulating survivin and blocking EGFR-ERK activation in renal cell carcinoma. Cancer Lett.

[17] Kim JS, Yang M, Lee CG, Kim SD, Kim JK, Yang K (2013). In vitro and in vivo protective effects of granulocyte colony-stimulating factor against radiation-induced intestinal injury. Arch Pharm Res.

[18] Jeong YJ, Jung MG, Son Y, Jang JH, Lee YJ, Kim SH, Ko YG, Lee YS, Lee HJ (2015). Coniferyl aldehyde attenuates radiation enteropathy by inhibiting cell death and promoting endothelial cell function. PLoS One.

[19] Wang J, Boerma M, Fu Q, Kulkarni A, Fink LM, Hauer-Jensen M (2007). Simvastatin ameliorates radiation enteropathy development after localized, fractionated irradiation by a protein C-independent mechanism. Int J Radiat Oncol Biol Phys.

[20] Moustakas A, Heldin CH (2016). Mechanisms of TGFβ-Induced Epithelial-Mesenchymal Transition. J Clin Med.

[21] Martin M, Lefaix JL, Pinton P, Crechet F, Daburon F (1993). Temporal modulation of TGF-beta 1 and beta-actin gene expression in pig skin and muscular fibrosis after ionizing radiation. Radiat Res.

[22] Randall K, Coggle JE (1996). Long-term expression of transforming growth factor TGF beta 1 in mouse skin after localized beta-irradiation. Int J Radiat Biol.

[23] Singh RP, Raina K, Deep G, Chan D, Agarwal R (2009). Silibinin suppresses growth of human prostate carcinoma PC-3 orthotopic xenograft via activation of extracellular signal-regulated kinase 1/2 and inhibition of signal transducers and activators of transcription signaling. Clin Cancer Res.

[24] Agarwal C, Singh RP, Dhanalakshmi S, Tyagi AK, Tecklenburg M, Sclafani RA, Agarwal R (2003). Silibinin upregulates the expression of cyclin-dependent kinase inhibitors and causes cell cycle arrest and apoptosis in human colon carcinoma HT-29 cells. Oncogene.

[25] Hoh C, Boocock D, Marczylo T, Singh R, Berry DP, Dennison AR, Hemingway D, Miller A, West K, Euden S, Garcea G, Farmer PB, Steward WP, Gescher AJ (2006). Pilot study of oral silibinin, a putative chemopreventive agent, in colorectal cancer patients: silibinin levels in plasma, colorectum, and liver and their pharmacodynamic consequences. Clin Cancer Res.

[26] Nambiar DK, Rajamani P, Deep G, Jain AK, Agarwal R, Singh RP (2015). Silibinin Preferentially Radiosensitizes Prostate Cancer by Inhibiting DNA Repair Signaling. Mol Cancer Ther.

[27] Yarnold J, Brotons MC (2010). Pathogenetic mechanisms in radiation fibrosis. Radiother Oncol.

