# Supplementary material for: Silibinin attenuates radiation-induced intestinal fibrosis and reverses epithelial-to-mesenchymal transition
Source: Oncotarget. 2017 Sep 2;8(41):69386–97. doi: 10.18632/oncotarget.20624 (PMC5642486; doi:10.18632/oncotarget.20624)
Supplement: Supplementary file 1 [file oncotarget-08-69386-s001.pdf]

Silibinin attenuates radiation-induced intestinal fibrosis and reverses epithelial-to-mesenchymal transition

SUPPLEMENTARY MATERIALS

Cell viability evaluation

Cell viability was evaluated using an MTT assay (Sigma-Aldrich, St. Louis, MO, USA). The assay is based on the reduction of tetrazolium dye MTT 3-(4,5-dimethylthiazol-2-yl)-2,5-diphenyltetrazolium bromide by living cells, which yields a soluble formazan product that can be detected colorimetrically. MTT solution was added to the cells in plates at 0.6 mg/ml in culture medium without FBS. The plates were incubated at 37°C for 2 h; then, the MTT solution was removed, and the cells were dissolved in a solution of DMSO. The solubilized formazan product was measured by an ELISA microplate reader at an absorbance of 560 nm. The data are shown as the mean±Standard Deviation (SD). Blank MTT levels were subtracted from the MTT values for each sample, and the results were normalized to 100%.

Cytotoxicity evaluation

Cytotoxicity was measured using a cytotoxicity assay kit from Biovision (Mountain View, CA, USA) in accordance with the manufacturer's recommendations. The assay was assessed quantitatively by the measurement of LDH (lactate dehydrogenase) release from damaged or destroyed cells into the extracellular fluid. LDH activity was monitored by the reduction of the tetrazolium salt (yellow) to formazan (red). Following 30 min of incubation in a 37°C incubator, the absorbance at 450 nm was measured. The data shown are mean±SD. Blank LDH levels were subtracted from the LDH values for each sample, and the results were normalized to 100%.

Measurement of apoptosis

Twenty female mice were divided into 4 groups (n=5 per group) as follows: a control group, a radiation group, and 2 groups that were administered silibinin. Silibinin (100 mg/kg) was administered to the mice orally 3 times with a 24 h interval before IR. The mice were sacrificed 12 h after IR (13 Gy) based on a report on the maximum number of apoptotic cells observed after radiation.19,20 Four-micron-thick sections were prepared and stained using hematoxylin and eosin (H&E) and the terminal deoxynucleotidyl transferase-mediated dUTP-biotin nick-end labeling (TUNEL) technique. Apoptosis in the paraffin sections was detected by TUNEL immunostaining using an ApopTag Plus™ kit (InterGen, USA) for analysis. Apoptotic cells in the longitudinal crypt sections showing a large portion of the crypt base, the lumen, and at least 17 cells along the crypt column were counted using an optical microscope (Nikon Eclipse 80i, Nikon Corporation). Cells were recorded as a single cell based on their size and clustering when several apoptotic fragments were believed to represent the remains of a single cell. Forty crypt sections were recorded for each mouse.

Jejunal crypt assay and morphological changes

Twenty-eight female C57BL/6 mice were divided into 4 groups (n=7 per group) as follows: a control group, a radiation group, and 2 groups that were administered silibinin. Silibinin was administered orally at 100 mg/kg for 6 consecutive days beginning 48 h prior to IR. The mice were sacrificed 3.5 days after IR. Their small intestines were fixed in 10% neutral-buffered formaldehyde and embedded in a paraplast wax to prepare 4-µm-thick tissue sections of the jejunum for H&E staining. Duplicate sections of 4 different parts of the jejunum from each animal were prepared for histological examination. The regenerating crypts in the jejunal cross-sections were then counted. To analyze morphological changes, all of the samples were sectioned and arranged into successive slices to search for the sample with the longest villi. This sample was used because it yielded more homogenous results than those of standard techniques based merely on the measurement of the 10 longest villi for a single slice of the sample. The lengths of the 10 longest villi, the crypts, and the basal lamina of 10

enterocytes from each sample were measured. Ten measurements were obtained for each animal, producing 50 measurements per group. Images of intestinal sections were obtained with a digital camera mounted on a Nikon Eclipse 80i microscope (Nikon Corporation). The quantification was performed using Image-Pro Plus image analysis software (Media Cybernetics, Bethesda, USA).

Colony formation assay

CT26 cells were plated into each 60-mm culture dish and allowed to grow following the Silibinin (10 and 20 μM) and IR (0–6 Gy) treatments at 37°C in a humidified 5% CO2 incubator. After 5 days, the dishes were stained with 0.4% crystal violet, and colonies (> 50 cells) were counted. The surviving fraction was calculated as the mean number of colonies/cells inoculated × the plating efficiency.

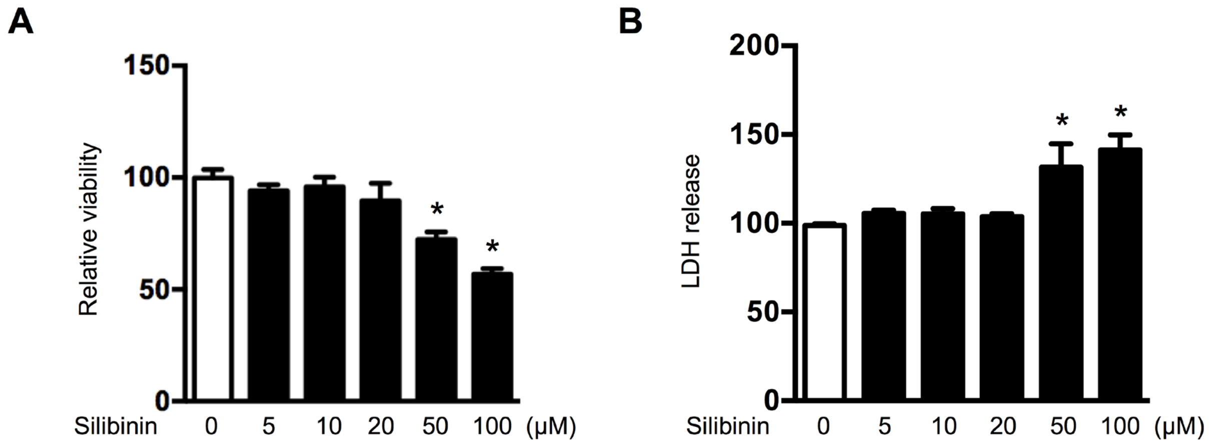

Supplementary Figure 1: The cytotoxicity and reversibility of radiation-induced EMT in IEC6 cells. (A) MTT assay and (B)LDH assay. The data are presented as the mean±SD; n=6, *p < 0.05 compared with 0 μM in each assay.

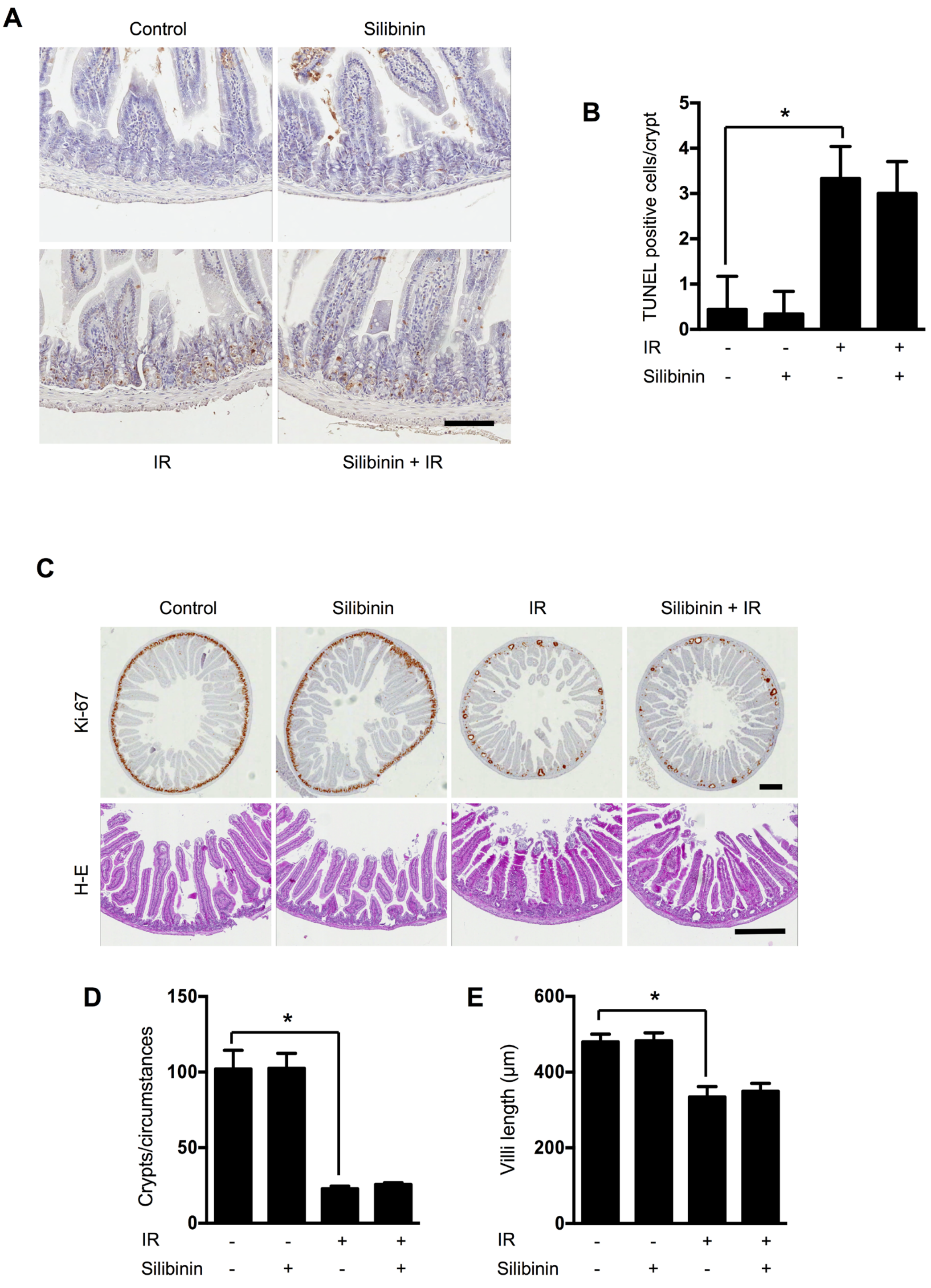

Supplementary Figure 2: Effects of silibinin on radiation-induced acute injuries in the intestine. (A) Representative images and (B) quantification of the apoptotic cell death at 12 h after exposure to 13 Gy of abdominal IR. Apoptosis in the jejunum was observed via TUNEL staining and counterstaining with hematoxylin (n=5, **p* < 0.05). Scale bar=100 μm. (C) Morphological changes in the intestine at 3.5 days after IR. H&E staining and immunohistochemistry for Ki-67, a proliferation marker, was conducted in sections of the jejunum. Ki-67 was detected using 3,3'-diaminobenzidine (brown) and hematoxylin counterstaining (blue). Scale bar=200 μm. (D) The numbers of crypt cells and (D) the lengths of villi were measured via H&E staining. The data are presented as the mean±SD; n=7, **p* < 0.05.

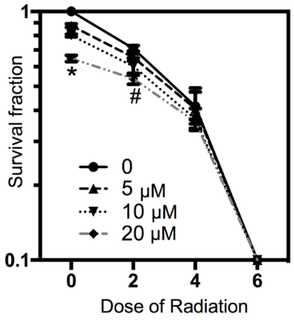

Supplementary Figure 3: Colony-forming assays were performed with CT26 cells. The cells were seeded and cultured for 7 days following silibinin (5-20 μM) and IR (2-6 Gy) treatment (mean±SD;n=3, *p < 0.05 compared with control, #p < 0.05 compared with 2 Gy irradiated control group).

Supplementary Table 1. Primer used in real-time RT-PCR analysis.

Gene	GenBank® accession number	Primer sequence		Tm (°C)	Product size (bp)
α-SMA	NM_007392.3	Forward	5'-CTGGAGAAGAGCTACGAACTGC-3'	61	368
		Reverse	5'-CTGATCCACATCTGCTGGAAGG-3'		
TGF-β1	NM_011577.2	Forward	5'-CTTCAGCTCCACAGAGAAGAACTGC -3'	64	299
		Reverse	5'- CACAATCATGTTGGGACAACTGCTCC -3'		
GAPDH	NM_008084.2	Forward	5′-TCCATGACAACTTTGGCATT-3′	55	377
		Reverse	5′-GTTGCTGTTGAAGTCGCAGG-3′		

Tm; melting temperature.
